# Bite of the Diamond Rattlesnake (Crotalus Adamanteus)*There are three species in the family—the *Diamond* rattlesnake, with the most poisonous virus of the North or South American continent; the *Banded* rattlesnake, whose virus is not quite so effective, but is destructive of human life; the *Ground* rattlesnake, of an inferior size, whose virus will not destroy human life, whose bite produces a chronic ailment, with pain and periodical swellings of the limb bitten, affected by transitions of temperature similar to that of a gun-shot wound.

**Published:** 1874-01

**Authors:** A. Mitchell

**Affiliations:** Portland


*Bite of the Diamond Rattlesnake* (*Crotalus Adamanteus*).* By A. Mitchell, M.D., Portland.

During my residence on the St. Illa river, in Southwest Georgia, on the 1st of April, 1864, I had occasion to attend a very severe bite of this venomous reptile, in the case of a colored boy, about fifteen years of age.

He was struck, about six inches above the external malleolus, on the outer edge of the gastrocnemius muscle, the leg being bared, and treading directly on the snake, while in the coil. The fangs entered deeply, inflicting a severe wound, when by his convulsive spring he tore them from his leg.

After receiving the bite, he ran about four hundred yards, and fell, in a convulsive tremor. The cries of his mother brought me to his side in ten minutes. I quickly applied tight ligatures above and below the knee, with firm compression over the popliteal region, and then made three incisions over the region of the wound, nearly an inch in depth, from which flowed freely a dark grumous blood. Large doses of carbonate of ammonia were freely administered, the bleeding encouraged by sponging the wound with warm water, and then the piston-cups were applied, and kept on for about forty minutes, until red arterial blood began to flow. My patient then becoming very weak, I withdrew the cups, after taking twenty ounces of blood. The pulse being depressed, with subsultus continuing, I administered two ounces of spiritus frumenti, with a little water, and had him removed to his home, and placed on his bed; the lower cord around the knee was then removed, and likewise the compression at the popliteal region.

Appearance of the patient much changed; great agitation, stupor, tremor, and prostration of the vital powers. Leg and thigh quite swollen; removed the upper ligature, applied pulvis nucis vomicæ to the wound, and enveloped the whole limb in a poultice composed of young fern, bruised, and saturated with a strong alkaline solution. Pulse 130, small in calibre; great thirst; skin cool; twitching of the muscles quite subsided, with the exception of some trembling of the muscles of the thigh; great pain in the region of the wound, and along the course of the nerves of the leg

* There are three species in the family—the *Diamond* rattlesnake, with the most poisonous virus of the North or South American continent; the *Banded* rattlesnake, whose virus is not quite so effective, but is destructive of human life; the *Ground* rattlesnake, of an inferior size, whose virus will not destroy human life, whose bite produces a chronic ailment, with pain and periodical swellings of the limb bitten, affected by transitions of temperature similar to that of a gun-shot wound.

and thigh; skin harsh and dry; ethereal anodyne administered; carbonate of ammonia continued in smaller doses; had a restless night.

2nd day.—Visited him early in the morning; found him feverish; pulse 120, and contracted; countenance anxious. Stupor continues, accompanied with depression of the nervous energies. Sensation of coldness over the whole body. Calls frequently for water, and rejects all nourishment. Slight twitching of the limb. Took three ounces of blood with the cups, just above the wound. Continued the alkaline poultice, with pulvis nucis vomicæ to the wound. Administered half a grain of podophyllin, with five grains of Dover's powder. Small doses of carbonate of ammonia continued. Ordered chicken broth; he swallowed a half-cupful with difficulty. Visited him in the afternoon. Leg and thigh much swollen to the hip-joint; bathed the limb with a strong decoction of arnica, and applied a firm roller, to be kept wet with the same. Visited him at 9 o'clock in the evening. Symptoms much the same, with sanious fluid escaping from the wound and smaller incisions. Fomented the limb with warm soap suds, and dressed with unguentum hydrargyri nitratis.

3rd day.—Visited him at day-break. Had some rest, from the ethereal anodyne; limb much swollen and sensitive to the touch. Scarified the thigh; a yellowish serous fluid escaped from these incisions. Pain quite abated. Continued the roller and bathing with arnica. Constitutional symptoms somewhat improved; stupor less; pulse more regular, slightly tremulous. Nothing having passed his bowels from the date of the injury, gave him an active cathartic, which produced a free bilious evacuation. Countenance, towards the close of the day, looks better; the pallor, shrinking of the features and sinking of the eye, improved; notices his dog; took some nourishment, the first he has taken since the bite, except the half-cup of chicken broth.

4th day.—Visited him in the morning. General appearances better; constitutional excitement abated, pulse nearly natural, little above the normal standard; swelling of the limb subsiding; perfectly conscious; yellowish serous fluid still oozing from the wounds. Roller and arnica continued, with simple dressings; gave him a dose of castor oil. No aggravated symptoms made their appearance afterwards; appetite returned, and he relished his food. On the 8th day, I allowed him to sit out-doors. He had a protracted convalescence, his recovery not being complete until the following month of September; a tonic was used, composed of tincture nux vomica and equal parts fluid ext. opium, twelve drops three times per day, with occasional use of the pills of podophyllin. This case presented an unusual symptom, as he would swell to such a degree, at stated periods, that his natural appearance was hardly recognizable; this quickly disappeared under simple treatment. Discharged, perfectly cured, the middle of September, 1864.

It will be seen that the boy was struck upon the bare surface, his trowsers being rolled above the knee, the fangs entering deeply with the poisonous virus, into a region where the absorbent vessels are distributed freely. The vital and chemical qualities of the blood, and its constituent properties, are almost instantaneously annihilated by the active conveyance of this virus through the absorbent system to the vital fluid. This boy was saved by the circumstance of my being on the spot directly after he received the bite. The cups and ligatures are hints from the aboriginal mode of treatment in like cases.—*Boston Med. and Surg. Journal.*

